# Supplementary material for: A model for the noninvasive, habitat-inclusive estimation of upper limit abundance for synanthropes, exemplified by M. fascicularis
Source: Sci Adv. 2024 May 24;10(21):eadn5390. doi: 10.1126/sciadv.adn5390 (PMC11122667; doi:10.1126/sciadv.adn5390)
Supplement: 20250207-1 [file sciadv.adn5390.v2.pdf]

ERRATUM PUBLISHED 7 FEBRUARY 2025; SEE LAST PAGE

ECOLOGY

A model for the noninvasive, habitat-inclusive estimation of upper limit abundance for synanthropes, exemplified by *M. fascicularis*

Copyright © 2024 The Authors, some rights reserved; exclusive licensee American Association for the Advancement of Science. No claim to original U.S. Government Works. Distributed under a Creative Commons Attribution License 4.0 (CC BY).

André L. Koch Liston[1,2,3]†, Xueying Zhu[3,4,5]†, Tran V. Bang[3,6], Phaivanh Phiapalath[3], Tanvir Ahmed[3,7,8], Sabit Hasan[3,9], Sajib Biswas[3,7], Shimul Nath[3,7], Toufique Ahmed[3,7], Kurnia Ilham[3,10,11], Ngwe Lwin[12], Jackson L. Frechette[13], Naven Hon[13], Cain Agger[14], Suzuki Ai[15,16], Emeline Auda[14], Eva Gazagne[17], Jan F. Kamler[18], Milou Groenenberg[19], Sarah Banet-Eugene[19], Neil Challis[3,20], Nicole Leroux[21], Pablo Sinovas[22], Vanessa H. Muñoz[23], Susan Lappan[24,25], Zaki Zainol[26], Valeria Albanese[3], Athanasia Alexiadou[3,4], Daniel R. K. Nielsen[3], Anna Holzner[3], Nadine Ruppert[3,25,26], Elodie F. Briefer[3,4], Agustin Fuentes[1,3], Malene F. Hansen[1,3,4,27]*

Accurately estimating population sizes for free-ranging animals through noninvasive methods, such as camera trap images, remains particularly limited by small datasets. To overcome this, we developed a flexible model for estimating upper limit populations and exemplified it by studying a group-living synanthrope, the long-tailed macaque (*Macaca fascicularis*). Habitat preference maps, based on environmental and GPS data, were generated with a maximum entropy model and combined with data obtained from camera traps, line transect distance sampling, and direct sightings to produce an expected number of individuals. The mapping between habitat preference and number of individuals was optimized through a tunable parameter ρ (inquisitiveness) that accounts for repeated observations of individuals. Benchmarking against published data highlights the high accuracy of the model. Overall, this approach combines citizen science with scientific observations and reveals the long-tailed macaque populations to be (up to 80%) smaller than expected. The model's flexibility makes it suitable for many species, providing a scalable, noninvasive tool for wildlife conservation.

INTRODUCTION

The accurate estimation of animal population sizes across large areas from camera trap data requires careful corrections to account for the lack of individual identification, the small extent of surveyed areas, and the limited size of datasets (*1*). It proves most effective for the study of large mammals (*2*), with extrapolations to larger regions from camera trap data alone intensifying the trade-off between dataset size and model accuracy. Tracking the movement of individually marked animals via mark-recapture or GPS tagging offers a solution but unavoidably disrupts the species' movement and behavior and requires substantial amounts of scientific resources and trained labor (*3*).

Currently, substantial strides are being made to circumvent these limitations (*4*), such as the integration of Bayesian computation and spatial-temporal information in population modeling, which offer ways to reduce bias across large bodies of data (*5*). When applied carefully to citizen science (*6*) or presence-only data (*7*), these treatments effectively enrich the total data available on a species for abundance estimation. Still, for species in which data are relatively scarce, such integration is not yet sufficient and the need remains for alternative approaches for assessing approximate population sizes from highly constrained datasets.

Without proper individual identification, the limited resolution of camera traps increases the likelihood of counting the same individual as two or more distinct individuals (double counting); even as machine learning models become more refined, differences in luminosity, weather, and camera angle can lead to unreliable results (*8*). These limitations are exacerbated by the small areas of separately surveyed regions (*9*), creating small and likely biased datasets that are generally not suitable for country-wide extrapolations. While recent methods attempt to overcome the limitations of individual identification, like the random encounter and staying time (REST) model (*10*), these approaches continue to fall short for estimates across larger regions if habitat diversity is not explicitly included in the analysis or if the distribution of camera traps is not random.

[1]Department of Anthropology, Princeton University, Princeton, NJ, USA. [2]Department of Chemistry, Columbia University, New York, NY, USA. [3]The Long-Tailed Macaque Project, Sorø, Denmark. [4]Behavioural Ecology Group, Department of Biology, University of Copenhagen, Copenhagen, Denmark. [5]School of Human Sciences, University of Western Australia, Perth, Australia. [6]Southern Institute of Ecology, Institute of Applied Material Science, Vietnam Academy of Science and Technology, Ho Chi Minh City, Vietnam. [7]Nature Conservation Management, Dhaka, Bangladesh. [8]Deutsches Primatenzentrum GmbH Leibniz-Institut für Primatenforschung, Göttingen, Germany. [9]Isabela Foundation, Dhaka, Bangladesh. [10]Museum of Zoology, Department of Biology, Andalas University, Padang, Indonesia. [11]Department of Biomedical Science and Environmental Biology, Kaohsiung Medical University, Kaohsiung, Taiwan. [12]Fauna & Flora International Myanmar, Yangon, Myanmar. [13]Conservation International, Phnom Penh, Cambodia. [14]Wildlife Conservation Society Cambodia, Phnom Penh, Cambodia. [15]Graduate School of Asian and African Area Studies, Kyoto University, Kyoto, Japan. [16]Open Innovation & Collaboration Research Organization, Ritsumeikan University, Kyoto, Japan. [17]Unit of Research SPHERES, University of Liège, Liège, Belgium. [18]Wildlife Conservation Research Unit, University of Oxford, Oxford, UK. [19]World Wide Fund for Nature Cambodia, Phnom Penh, Cambodia. [20]Neil Challis Photography, Kanchanaburi, Thailand. [21]Wildlife Alliance, Phnom Penh, Cambodia. [22]Fauna & Flora International Cambodia, Phnom Penh, Cambodia. [23]Fishing Cat Ecological Enterprise Co. Ltd., Phnom Penh, Cambodia. [24]Department of Anthropology, Appalachian State University, Boone, NC, USA. [25]Malaysian Primatological Society, Kulim, Malaysia. [26]School of Biological Sciences, Universiti Sains Malaysia, Pulau Pinang, Malaysia. [27]Wildlife Trade Research Group, Oxford Brookes University, Oxford, UK.
*Corresponding author. Email: malenefriishansen@gmail.com
†These authors contributed equally to this work.

Camera trap surveys, drone surveys, and the use of citizen science data provide a cost- and labor-efficient way of surveying larger areas and producing GPS locations that can be used for population estimation. Distance sampling has long been a favorable method for estimating primate density and abundance but remains highly labor intensive and requires in-depth knowledge of the region, limiting its application to small areas (*11*, *12*). Furthermore, several assumptions, such as randomization and replication, must be met for reliable population size inference (*13*).

Many current efforts of noninvasive sampling focus either on arboreal species, by infrared drone imaging, or on solitary species, like most camera trap efforts, making camera traps alone less efficient in characterizing group-living synanthropes when the size of the datasets is small. Although Bayesian modeling can offer an effective solution to increase datasets by combining different data sources (*14*), this is most reliable when the region investigated is constant for the different methods of data collection (*15*). As human-wildlife interfaces increase, more animal species are bound to exhibit synanthropic patterns, highlighting the need for tools to effectively estimate their populations sizes accurately.

In this study, we developed a noninvasive probability model to estimate the maximum population abundance of large-bodied semi-terrestrial/terrestrial group-living animals. Probabilities for group sizes are assigned according to the number of individual animals observed using a log-normal distribution combined with a tunable variable (*16*). The model was specifically applied to the long-tailed macaque (*Macaca fascicularis*), a nonhuman primate in need of an updated population size estimate (*17*, *18*). Starting from a first-principles description of animal behavior, a probability model was empirically corrected by camera trap and sighting data, as well as information on species behavior and habitat preference (*19*). This involved combining a probability description with the explicit inclusion of variable parameters (e.g., species-species demography and habitat preference), aiming to accurately replicate the population abundance for fully mapped areas (*20*). This offers a flexible and transparent method to estimate maximum population abundance across larger regions: By starting from a first-principles analysis and openly incorporating species behavior—along with its inherent uncertainty and regional intraspecific variation—the model relies on a small set of variables that may be tailored to different species or particular regions.

The long-tailed macaque is widely known for its synanthropic behavior, wide range, and perceived ubiquity, often resulting in the assumption of overabundance (*21*, *22*). However, only a few attempts have been made to accurately estimate its population size (*23*–*25*). *M. fascicularis* is distributed from the northern coastal areas of the Rakhine including the southeastern coast of Bangladesh to the southern reaches of Indonesia and the eastern Philippines islands (*23*), although recent findings indicate extirpation in Bangladesh and in local areas of Cambodia and Laos (*26*). This generalist primate displays synanthropic versatility across various habitats, including forests, grasslands, coastal areas, agricultural lands, urban areas, and temples, and is able to use human resources (*24*, *27*). Despite substantial overlap between long-tailed macaques and humans and subsequent assertions of overabundance, recent research suggests that the long-tailed macaque population is undergoing a persistent decline. This is partially due to the overlap with humans and thereby visibility and vulnerability to threats, such as hunting and culling, and partially due to the demand for the species for the pet trade, meat trade, entertainment, and trade for biomedical and pharmaceutical research (*17*, *18*, *22*, *26*). The amount and severity of threats led to the 2022 IUCN Red List listing of the species as Endangered A3cd (*18*). The IUCN Red List classification of *M. fascicularis* is currently being contested (*18*, *28*). This and the factors outlined above make it an interesting test subject for our model, enabling us to simultaneously test the model and provide crucial information for the conservation of the species.

RESULTS

Habitat preference

The area under the receiver operating characteristic curve (AUC) value for the test data was 0.854, while the AUC value for the training data was 0.870, showing good model performance (fig. S1). The omission rate of the test and training data was essentially consistent with the predicted omission rate, indicating the absence of spatial autocorrelation and a good modeling effect (fig. S2) (*29*). The combined evaluation of AUC values and omission rates suggested that the model predictions have high accuracy and reliability. Figure 1 shows the habitat preference map for the long-tailed macaque with all environmental variables and suitability, values ranging from 0 to 1.

Maximum abundance estimation

The habitat preference–population curves primarily exhibit an increasing trend, with the curve that replicates the best estimation for the population at Keo Seima ($\rho_{sanctuary}$) (*20*) located between the lower and upper bounds, ρ_{lower} and ρ_{upper}, respectively (Fig. 2). Likewise, these curves approximate scaled versions of each other, in alignment with the expected influence of inquisitiveness ρ on abundance estimations.

When applied to the habitat preference map of Keo Seima Wildlife Sanctuary (KSWS), these curves correctly reproduce the reported populations of 854, 1566, and 3097 for lower, best, and upper ρ estimates, respectively. When applied to the habitat preference map of Cát Tiên National Park (CTNP)—whose observation data were not directly used in any of the models—the estimations closely match the predictions from direct observations: 255, 509, and 1009 for lower, best, and upper ρ values, respectively.

Considering that each spot in the model has a fixed area of 1.143 km^2, the average of each habitat preference–population curve (1.052, 0.530, and 0.267 individuals per spot) can be used to approximate an average density of long-tailed macaque groups across large regions, namely, 0.921, 0.464, and 0.233 groups/km^2 for each increasing value of ρ. These results agree remarkably well with reported group densities in the literature, ranging from 0.6 to 1.3 groups/km^2 (*30*).

Upon extrapolation to the entire habitat preference map (wherein not all regions are protected areas), these curves produce a best estimate, as well as the range extremes, for the upper limit population of long-tailed macaque individuals in each country and across the entire region (Table 1). The model draws most data from Cambodia, Laos, Malaysia, and Vietnam and was parameterized against KSWS (within Cambodia) and was tested against CTNP (within Vietnam). Therefore, predictions are expected to be most accurate within these countries ("high confidence countries"; fig. S3A). The model's generality allows extrapolation to other Southeast Asian countries, albeit with lower accuracy ("lower confidence countries"; fig. S3B).

ERRATUM PUBLISHED 7 FEBRUARY 2025; SEE LAST PAGE

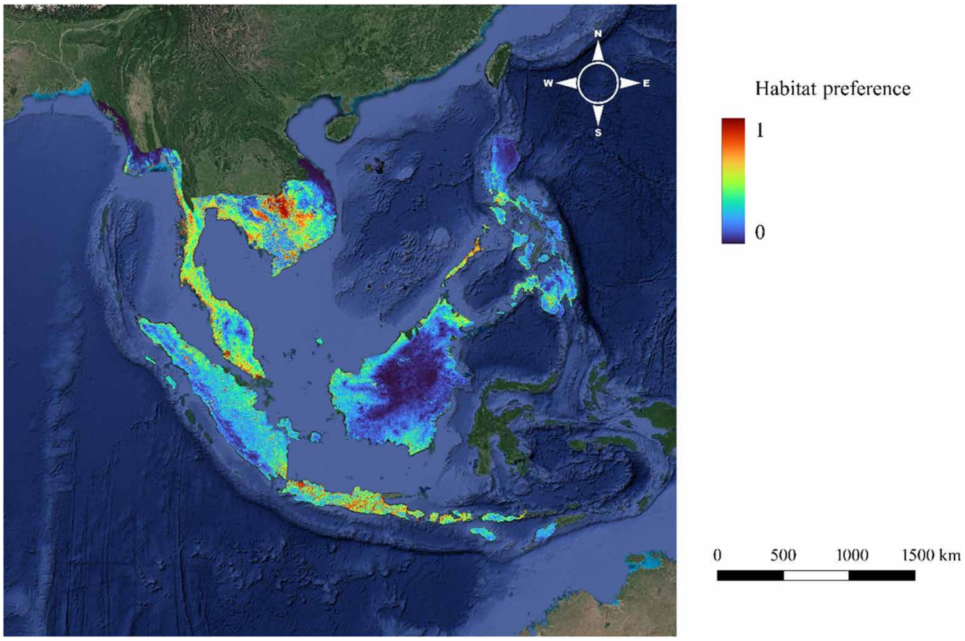

Fig. 1. Habitat preference map for *M. fascicularis* in Cambodia, Indonesia, Laos, Malaysia, Myanmar, Philippines, Singapore, Thailand, and Vietnam. Red and blue colors indicate a high and low habitat preference, respectively, thus suggesting a greater and smaller likelihood of. *M. fascicularis* inhabiting the area.

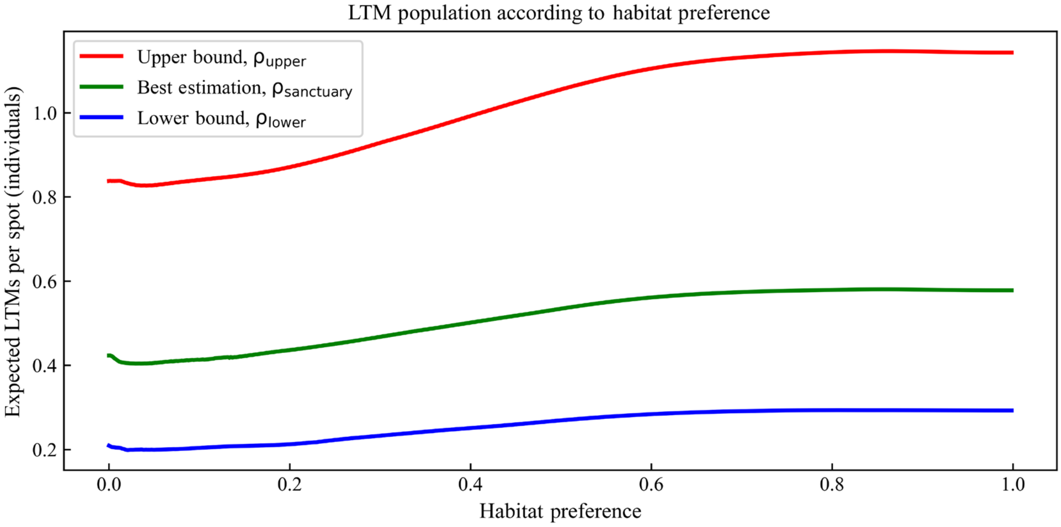

Fig. 2. Mean habitat preference versus population curves in a protected wildlife sanctuary, $\rho_{sanctuary}$. LTM, long-tailed macaque.

DISCUSSION

Our aim was to develop a noninvasive probability model to estimate the maximum population abundance of large-bodied semiterrestrial/terrestrial group-living animals, hoping to overcome current problematics surrounding abundance estimation from camera trap and direct sighting data. This method for estimating the upper limit abundance of a species' population is both flexible and robust: It eases the inclusion of species-specific data to accommodate a growing body of knowledge while relying on benchmarked results to ensure model accuracy. The model is not strictly limited to a single species. Its parameters, explicitly derived from information on the species, can be adjusted to approximate a wide range of wildlife populations in a noninvasive manner. It is important to note that the calibration of this application of the model currently relies on a single site, KSWS, Cambodia. Biases in the population estimation for this region (*31*) are bound to be magnified in the extrapolation. Nevertheless, most biases tend to contribute to the underestimation of densities, with a smaller number of observed groups and individuals (*11*), thereby reiterating the upper limit estimation of this approach. Our approach includes different data collection methods, and this can lead to unknown errors and difficulties with comparisons over time (*12*); however, given the difficulties of obtaining data with time, labor, and funding constraints, one can argue that it is crucial to use the data available to develop initial assessments and our method provides this opportunity.

ERRATUM PUBLISHED 7 FEBRUARY 2025; SEE LAST PAGE

Table 1. Estimate for upper limit long-tailed macaque populations in Southeast Asia, extrapolating behavior for the protected area of KSWS, $\rho_{sanctuary} = 0.523813$.

Location	Best upper limit estimation	Extremes of upper limit estimation
High confidence countries	269,929 ± 520	134,320–538,525
Cambodia	75,544 ± 275	37,768–150,142
Laos	10,933 ± 105*	5,439–21,774
Malaysia	119,499 ± 105	59,319–238,905
Vietnam	63,953 ± 253	31,794–127,704
Lower confidence countries	632,709 ± 795	313,614–1,266,924
Indonesia	428,404 ± 655	212,063–858,805
Myanmar	42,239 ± 206	21,016–84,571
Philippines	102,594 ± 320	50,756–205,671
Singapore	234 ± 15	119–463
Thailand	59,238 ± 243	29,660–117,414
Entire region	904,638 ± 1,210	448,941–1,809,400

*A thorough investigation from 2021 to 2023 only revealed approximately 600 individuals in entire Laos (P. Phiapalath, personal observation).

Our research provides an important perspective on the population trends of the long-tailed macaque and illustrates the applicability of our model. In this assessment, we reasonably estimated the population size and group density for a social, primarily ground-dwelling synanthrope. We outlined a noninvasive tool for conservation efforts of underrepresented species.

Fooden's assessments of the long-tailed macaque population size from 1990 to 2006 indicated a decrease from 5 million to 3 million individuals (*25*, *32*). Our current estimate of approximately 1 million reflects a continuous decline representing an alarming 80% reduction over approximately 35 years (*33*). The severity of this decline is further emphasized by the nature of the model, which overestimates the population due to its calibration in a protected area, making the true decline possibly greater. Despite the methodological differences preventing direct comparison across all regions and sites, this decline raises concerns. While the numbers of long-tailed macaques might increase locally at points across their range, especially at interfaces with human settlements (*34*), our analysis suggests that these local increases do not reflect overall trends across Southeast Asia. The perception of long-tailed macaque ubiquity likely results from a selection bias, whereby degraded habitats force group migration closer to human settlement, where the species is more likely to be observed and reported.

Despite this model's relative success in providing reasonable estimates for long-tailed macaque populations, discrepancies may exist on the ground due to a variety of factors. For instance, while our estimates for Laos are much higher than the 300 to 600 individuals reported by ground observations (*35*) (P. Phiapalath, personal observation), the lack of protection from hunting for the species implies that even areas deemed suitable habitats may not accurately reflect the true population numbers. Furthermore, given the substantial and rapid decline we report here, reductions may occur faster than our data collection efforts, despite our use of recently collected data (2020–2022). On the other hand, a 2015 report from Cambodia, based on data from 2009, estimated 3 million free-ranging long-tailed macaques (*36*), and if this is accurate, it indicates a drastic 97.5% decline over 14 years when compared to our current findings. Although the 3 million population size estimate by the Cambodian government does not correlate well with Fooden's estimates of the same number for the entire species' range, a decline in population size is evident in Cambodia. Considering that we provide an upper limit estimate for Cambodia, we are concerned that the actual population size may be even lower.

Our results for Malaysia indicate a lower estimate compared to a previous survey in 2011, where the population size of mainland Malaysia was estimated at 127,050 individuals (*37*). Our estimate for entire Malaysia is 119,499, suggesting a reduction. However, we cannot estimate the percentage decline as no population size for Bornean Malaysia was provided in 2011. In Malaysia alone, 493,823 individual long-tailed macaques were reported culled from 2011 to 2018, primarily due to assessments categorizing them as conflict macaques (*38*). Should the reported culling amount be accurate, the population has fluctuated over time and now experienced a substantial reduction warranting a reevaluation of culling amounts and strategies.

Our estimate for Vietnam is the first nationwide estimation for the country and could be used as a baseline for future assessments and to inform conservation efforts. However, we caution against relying exclusively on this upper limit estimate as ground surveys suggest that the true population size might be lower (T. V. Bang, personal observation). Overall, we provided lower confidence estimates for Indonesia, Myanmar, Philippines, Thailand, and Singapore. Nevertheless, the model remains able to estimate population sizes across areas where direct data are not available. Hence, even for so-called lower confidence countries, our findings can serve as baseline estimates assisting in conservation management activities.

In this case study, using the long-tailed macaque, a notable drawback is the output of an upper limit for the species' population constrained by benchmarking to regions with abundant data. This limitation forces the model's optimization for protected areas, originating from the KSWS and CTNP. These areas prohibit predatory hunting and have relatively effective regulations. The same does not hold true for the entire region to which the model was extrapolated, inevitably overestimating the number of individuals in these areas.

Therefore, the numbers reported herein must be considered as an upper limit abundance estimation for the species in question, although groups sizes for provisioned populations are included. Moreover, the abundance estimation model strongly relies on averages across large regions scaled to match the true population inferred from direct observations. Hence, error cancellation is more pronounced in larger study areas. As a result, population estimates for larger countries (e.g., Vietnam) or regions (e.g., the Greater Mekong) are invariably more accurate than those for individual, smaller regions (e.g., Singapore).

In addition, our long-tailed macaque presence records, sourced from various channels including citizen science platforms, lack expert verification, posing a risk of misidentification, especially concerning hybrids with other macaque species occurring across some of the *M. fascicularis* range. The presence data also exhibited a lack of distribution, evident in the absence of observations in some inland areas. Hence, a comprehensive, long-term monitoring approach, considering factors influencing population dynamics, is essential for accurate models. Adopting such an approach is necessary for ensuring model accuracy by closely tracking changes in populations and factors influencing population dynamics.

In conclusion, in our model, we have identified a sustained decline in the population of long-tailed macaques, consistent with observed trends across recent decades. We believe that the population estimates produced here merit use in conservation measures as baseline abundance estimates (*17*, *22*). Population estimates for long-tailed macaques have been requested by international monitoring and conservation agencies for decades. Our results are the first to enable comparison across countries and regions, highlighting their suitability for informing conservation action. Our best upper limit population estimates coupled with our habitat preference map can inform national and local conservation measures, CITES (Convention on International Trade in Endangered Species of Wild Fauna and Flora) management efforts, and IUCN guidelines moving forward. Last, our model, when adapted to other species, holds potential in contributing to biodiversity conservation using both current and previous data for various species in the years to come.

MATERIALS AND METHODS

The method has been fitted to the ODMAP (Overview, Data, Model, Assessment and Prediction) protocol database and is available in the Supplementary Materials, offering a breakdown of the model's key steps to ease understanding and reproducibility (*39*).

Study area

Our study area extends from Bangladesh (Cox's Bazar: 21°25′38.0208″N and 92°0′20.9052″E) through the Greater Mekong into mainland Malaysia to Indonesia (Flores: −5°36′59.99″S and 119°29′59.99″E) and the Philippines (Luzon: 16°33′58.4388″N and 121°15′45.4824″E), including almost the entire native range of the long-tailed macaque. Habitat survey included forests, savannahs, and mangroves in protected national parks, as well as mixed secondary habitats, coastal lines, and forest edges in urban landscapes.

Data collection

Presence data of free-ranging long-tailed macaques were collected from multiple sources in Cambodia, Indonesia, Laos, Malaysia, Myanmar, Philippines, Singapore, Thailand, and Vietnam from January 2020 to December 2022 (table S1). An extensive survey took place in southeastern Bangladesh during the study period, yet no individuals were detected. Data sources included camera traps, line transect distance sampling sightings, and direct sightings, which represent 33.18, 1.19, and 65.63% of GPS location records, respectively. A total of 3077 location records were used in the habitat preference modeling, each record capturing one or more individual sightings.

Distribution and behavior of long-tailed macaques are influenced by multiple factors, such as topography, habitat type, anthropogenic disturbances, and food and water sources. These factors can be broadly categorized into four major classes: bioclimatic, geographical, land cover, and interference variables. Bioclimatic variables reflect seasonal variations in temperature and rainfall, as well as their potential impact on macaque behavior and food availability (*40*). Among the 19 climate factors in WorldClim, including 11 temperature-related and 8 precipitation-related factors, principal components analysis and correlation were used to screening to identify the six most important bioclimatic factors (figs. S4 and S5) (*41*). This approach aimed to avoid excessive similarity among factors that could lead to multicollinearity in the maximum entropy (MaxEnt) model. Geographical variables, including elevation, aspect, and slope, can indirectly influence the macroclimate, microclimate, and vegetation types (*42*). Slope and aspect were extracted using the digital elevation model data from NASA Earthdata and Japan Space Systems (*43*), and they were processed using ArcGIS Pro spatial analysis tools. The land cover variables encompassed built areas, crop lands, grasslands, shrublands, and forests, providing insights into the terrestrial vegetation types and anthropogenic modification. Considering *M. fascicularis*' frequent proximity to water resources and human habitats (including roads) (*13*, *25*, *44*), we included two interference variables: distance to the coastline and distance to roads. On the basis of the geological and ecological patterns in Southeast Asia and usages in previous research, this study selected a total of 16 environmental factors for the modeling of long-tailed macaques (Table 2).

The environmental data underwent a consistent projection process in both ArcGIS Pro and QGIS 3.32.0, ensuring uniform layer boundaries, coordinates, and grid sizes. Subsequently, all data files were converted into ASCII (ASC) format. These variables were used in the development of a habitat preference model for *M. fascicularis* across the regions of Cambodia, Indonesia, Laos, Malaysia, Myanmar, Philippines, Singapore, Thailand, and Vietnam.

Data analysis

Model summary

In general, the model works by replicating abundance estimates for group-living animals within small, mapped areas using a corrected first-principles probability approach. The correction incorporates experimental GPS signals (s) to establish a correlation with habitat preference (h) for estimating the upper limit of the population across larger areas. Experimental GPS signals (s) represent the reported number of individuals equal to or greater than 1 for all data points. The number of observed GPS signals (s) is related to a probability distribution for the number of individuals in the group responsible for these signals—that is, $P\{N = n|S = s\}$. These probability distributions are optimized by fitting a tunable variable (ρ) to experimental data. These corrected probability distributions generate an expectation value for the group size as a function of the number of GPS signals ($<N|S = s>$), namely

Table 2. Environmental variables required for long-tailed macaque habitat preference modeling.

Categories	Factors	Descriptions	Source
Bioclimatic variables	Bio1 (°C)	Annual mean temperature	WorldClim, v 2.1
	Bio3	Isothermality	
	Bio4 (°C)	Temperature seasonality	
	Bio8 (°C)	Mean temperature of wettest quarter	
	Bio13 (mm)	Precipitation of wettest month	
	Bio15 (mm)	Precipitation seasonality	
Geographical variables	Elevation (m)	Elevation	EarthEnv, Topography
	Slope (°/100)	Slope	Advanced Spaceborne Thermal Emission and Reflection Radiometer (ASTER)
	Aspect (°)	Aspect	
Land cover variables	Built (%)	Fractional cover for the built-up class	Copernicus Global Land Service: land cover 100 m, collection 3, epoch 2019
	Crop (%)	Fractional cover for the cropland class	
	Grass (%)	Fractional cover for the herbaceous vegetation class	
	Shrub (%)	Fractional cover for the shrubland class	
	Tree (%)	Fractional cover for the forest class	
Interference variables	Dist_coast (m)	Distance to the coastline	Natural Earth, 10 m Coastline
	Dist_road (m)	Distance to the road	Socioeconomic Data and Applications Center, Global Roads

$$E_N(s) = \langle N|S=s\rangle = \sum_{N_{min}}^{N_{max}} \left(n \cdot P\{N=n|S=s\}\right) \quad (1)$$

These expectation values are assigned to fixed area spots according to their corresponding geographical coordinates (rectangular regions spanning 0.0131 degrees in latitude and longitude; 1.033 km by 1.107 km north-south and east-west, respectively, for a fixed area of 1.143 km^2). The habitat preference for these spots is derived from the habitat preference map as an input that approximates favored areas for a particular species based on environmental factors (altitude, forest density, and water proximity). This correlation between the expected number of individuals per spot and habitat preference is then extrapolated to larger areas, generating a habitat-inclusive estimation of the maximum number of individuals within a region.

MaxEnt modeling

We used a MaxEnt modeling methodology to map the habitat preference of *M. fascicularis* (*45*), considering the influence of a total of 16 environmental variables (Table 2). MaxEnt aims to estimate a target probability distribution by finding the probability distribution of MaxEnt, requiring only presence and environmental data (*46*). We supplied the presence of localities as GPS points of free-ranging long-tailed macaques in Cambodia, Indonesia, Laos, Malaysia, Myanmar, Philippines, Singapore, Thailand, and Vietnam. The geospatial coordinates, encompassing longitudes and latitudes, of all observed long-tailed macaque presence locations were transformed into CSV format. Simultaneously, environmental variable datasets were converted into ASC format. The dataset was divided, assigning 75% of the records for training and allocating the remaining 25% for model testing (*45*). We set the "apply threshold rule" to "10 percentile training presence" and retained default values for other parameters. The output format specified as "cloglog" provides probability estimates of presence ranging between 0 and 1. We used the AUC and the omission rate to test the accuracy of the model prediction. A larger AUC indicates better performance; typically, an AUC value between 0.7 and 1.0 suggests moderate to high performance (*47*). Meanwhile, we assessed the matching between the omission rate in training and test presence records and the predicted omission rate, where higher matching indicates more accurate model predictions (*29*).

Upper limit abundance estimation

Raw data of GPS signals and model inputs

The geographical GPS coordinates corresponding to long-tailed macaques' observations from camera traps, line transects, or reported sightings were grouped together according to home range: GPS signals were grouped when closer than 800 m = $(2/\pi\ \text{km}^2)^{1/2}$ = the radius of a 2-km^2 circle (approximate upper bound home range of a long-tailed macaque group) (*48*).

The semiempirical probability model includes eight species-specific input parameters (Table 3), one of which (ρ) encapsulates the uncertainty in individual long-tailed macaque behavior and is thus continuously varied to match experimental measurements. N_{min} and N_{max} define the bounds for the minimum and maximum number of individuals in a long-tailed macaque group, respectively. These were collected by combining a literature review with personal observations from the authors. Because of the distinctive synanthropic behavior of *M. fascicularis*, group size varies greatly between nonprovisioned and provisioned groups (*49*). Provisioned ratio captures this variation as the ratio of provisioned to nonprovisioned groups in the species. $N_{mean}^{[1]}$ and $N_{mean}^{[2]}$ denote the mean number of individuals in a nonprovisioned ([1]) and provisioned ([2]) group, respectively. $\sigma_{sd}^{[1]}$ and $\sigma_{sd}^{[2]}$ denote the SD of $N_{mean}^{[1]}$ and

Table 3. Summary, significance, and numerical value of input parameters for model.

Variable	Significance	Value (long-tailed macaques)
N_{min}	Smallest number of individuals in a group	5
N_{max}	Largest number of individuals in a group	200
ProvRt	"Provisioned ratio," the proportion of provisioned to nonprovisioned groups across long-tailed macaque populations	0.025
$N_{mean}^{[1]}$	Mean number of individuals in a nonprovisioned group	20
$N_{mean}^{[2]}$	Mean number of individuals in a provisioned group	170
$\sigma_{sd}^{[1]}$	SD ($\sqrt{}$Variance) of individuals in nonprovisioned groups	15
$\sigma_{sd}^{[2]}$	SD ($\sqrt{}$Variance) of individuals in provisioned groups	30
ρ	Inquisitiveness factor of species. Describes the tendency of individuals to be registered as GPS signals more than once. Varies continuously from a lower to an upper bound (0 to 1), optimized by an empirical fit.	{0, 0.523813, 1}

$N_{mean}^{[2]}$, respectively. The variable ρ (termed as "inquisitiveness") represents the likelihood of individuals within the species being registered as a GPS signal more than once, resulting in double counting. It is intuitively proportional to the tendency of an individual to return to a GPS detection source and be observed more than once and inversely proportional to the actual group size.

Probability model

The probability matrix $P\{N = n|S = s\}$ is obtained semiempirically, supplementing a first-principles derivation with experimental data on the species.

For a specific geographical location (spot), $S = s$ is the number of GPS signals observed and $N = n$ is the number of long-tailed macaque individuals in the group located at that specific spot. Assuming that all individuals in the group are equally likely to be detected in GPS signals, there are n^s possible ways of obtaining $S = s$ GPS signals. For instance, consider $N = 7$ individuals in a spot; the number of ways to generate $S = 3$ is then 7*7*7 = 73 = 273. Likewise, for $N = 10$ individuals, the number of ways is $10^3 = 1000$.

Since group sizes are not limitless, the number of individuals is bound between N_{min} and N_{max}. Hence, in this simplified analysis, the normalized matrix becomes

$$P\{N = n|S = s\} = \frac{n^s}{\sum_{N_{min}}^{N_{max}} (n^s)} \tag{2}$$

The lack of individual identification within most datasets might lead to double counting of individuals. To address this, repetitions of GPS signals are treated as "dead signals," representing observations from previously identified individuals. Hence, the realistic number of GPS signals is obtained by omitting these repetitions.

Because individual identification remains unreliable for long-tailed macaque camera trap data (among many other species) despite advances in artificial intelligence (*50*), we introduce an empirical exponential correction (ρ) to the number of GPS signals (s) so that $\sigma = s^{(1-\rho)}$. It represents the number of GPS signals adjusted for double counting in each geographical spot. The continuous variable $\rho \in [0, 1]$ is equivalent to an inquisitiveness factor due to its effect on the adjusted number of GPS signals (σ). When $\rho = 0$, individual long-tailed macaques are fully noninquisitive: Every reported GPS signal (in the same spot) is treated as a unique GPS signal from nonrepeated individuals ($\sigma = s$). When $\rho = 1$, long-tailed macaques are fully inquisitive: All reported GPS signals (in the same spot) are treated as repeated GPS signals from the same individual ($\sigma = 1$). Thus, the semiempirical probability matrix becomes

$$P\{N = n|S = s\} = \frac{n^{s^{(1-\rho)}}}{\sum_{N_{min}}^{N_{max}} \left[n^{s^{(1-\rho)}}\right]} \tag{3}$$

The final empirical correction to the probability model comes from the base probability distribution for $P\{N = n\}$, known to be nonuniform. We account for the split in long-tailed macaque groups between nonprovisioned ([1]) and provisioned ([2]) environments by normalizing two united log-normal distributions (LNorm) (*51*). With this as the base correction to the group size distribution, the final probability matrix becomes

$$P\{N = n|S = s\} = \frac{\mathrm{LNorm}_1(n) \cdot \mathrm{LNorm}_2(n) \cdot \left[n^{s^{(1-\rho)}}\right]}{\sum_{N_{min}}^{N_{max}} [\mathrm{LNorm}_1(n) \cdot \mathrm{LNorm}_2(n)] \cdot \sum_{N_{min}}^{N_{max}} \left[n^{s^{(1-\rho)}}\right]} \tag{4}$$

where

$$\mathrm{LNorm}_i\left(n, \sigma_{sd}^{[i]}, N_{mean}^{[i]}\right) = \left\{ \frac{(n \bullet Z^{[i]} \bullet \sqrt{2\pi})^{-1} \cdot e^{-[\ln(n) - M^{[i]}]^2}}{2(Z^{[i]})^2} \right\}^{\left(\frac{1}{\mathrm{ProvRt}}\right)}$$

$$Z^{[i]}\left(\sigma_{sd}^{[i]}, N_{mean}^{[i]}\right) = \sqrt{\ln\left[1 + \left(\frac{\sigma_{sd}^{[i]}}{N_{mean}^{[i]}}\right)^2\right]}$$

$$M^{[i]}\left(\sigma_{sd}^{[i]}, N_{mean}^{[i]}\right) = \ln\left[\frac{\left(N_{mean}^{[i]}\right)^2}{\sqrt{\left(N_{mean}^{[i]}\right)^2 + \left(\sigma_{sd}^{[i]}\right)^2}}\right]$$

For a small number of GPS signals ($s < 45$), the probability of a small (nonprovisioned) group size, $N \in \{5, 20\}$, dominates the distribution (Fig. 3). As the number of GPS signals increases ($s > 45$), the probability distribution shifts to favor a large (provisioned) group size, $N \in \{150, 200\}$. As the number of GPS signals approaches infinity ($s \to \infty$), the entire probability distribution concentrates at $N_{max} = 200$ since $\lim_{s\to\infty} P\{N = N_{max}|S = s\} = 1$.

These probability distributions lead to a sinusoidal behavior in the expectation value of N for a given S, $E_N(s) = <N|S = s>$ (Fig. 4).

$E_N(s)$ is a strictly increasing function whose lower and upper bounds approach the mean group size, N_{mean}, for nonprovisioned and provisioned groups, respectively. This relationship enables the determination of an expected number of individual long-tailed macaques for any given number of GPS signals.

Benchmarking against KSWS (Cambodia) and comparison with CTNP (Vietnam)

To ensure the accuracy of the probability model and optimize the empirical inquisitiveness factor ρ, the population estimate was benchmarked against published data for the KSWS (*10*). For this area, both camera trap data and a population range estimate were available. By monitoring 40 line transects from 2010 to 2020, the previous distance sampling (*20*) provides a best estimate from 2020 of 1566 individual long-tailed macaques in the region, with lower and upper bounds of 792 and 3097, respectively. Camera trap data for Keo Seima (obtained through the Wildlife Conservation Society, from 2020 to 2022) total 960 GPS signals spread across 82 nonoverlapping spots (coordinates separated by more than 800 m); an estimated population of 1566 individuals is obtained under an optimal $\rho_{sanctuary} = 0.523813$.

For this $\rho_{sanctuary}$, every distinct individual is responsible for approximately three camera trap signals (about two of every three GPS signals are considered to be repetitions). The upper bound of 3097 is appropriately replicated for $\rho_{upper} = 1$ (for which every GPS signal from every spot is treated as representing a singular individual); the lower bound of 792 is closely matched, though slightly overestimated by 8%, for $\rho_{lower} = 0$ (for which all GPS signals from the same spot are treated as repetitions). These three inquisitiveness factors (ρ_{lower}, $\rho_{sanctuary}$, and ρ_{upper}, tailored for the protected area of a wildlife sanctuary) are used to generate three different $E_N(s)$ curves, similar to the one reported above. As a brief check of model accuracy, these three parameters were applied to observations from CTNP, yielding results in a reasonable order of magnitude. Table S2 summarizes these results for the Keo Seima and Cát Tiên protected areas.

It is important to note that this optimization relies on a protected area, in which individuals are supposedly shielded from human predatory pressures. As a result, this model—in this particular case study of *M. fascicularis*—implicitly treats all regions as protected areas. Since this cannot be true for all regions in all countries across Southeast Asia, the results of our model for this species offer a high upper limit for population abundance. Moreover, the calibration of the model assumes the previous assessment (*20*) to be accurate, implying that biases in the sampling method used invariably translate to the current exemplification of the model.

The greatest challenge in correlating habitat preference (h) to the expected number of individuals ($E_N = <N>$) is the null result penalty: Locations where groups of a given species have not been observed go unreported by researchers and receive little to no further investigation. In the attempt to account for absence data and, more accurately, relate h to E_N, a diffusion factor based on the cardinality of habitat preference values in the map and in the data signal spots was used. The raw diffusion factor quantifies the total number of pixel spots in the map that share habitat preference with the data signal (Fig. 5). Our analysis focuses on different regions: the entire habitat preference map, revealing a scarcity of pixels with a habitat preference greater than 0.8 and an abundance below 0.2; the KSWS, which contains mostly pixels with a habitat preference above 0.6, indicating the likely presence of long-tailed macaques in the area; and the CTNP, showing a more Gaussian spread in habitat preference values, making it appropriate for evaluating model consistency.

These raw diffusion factors (one for each value of h) are then subject to an average mean filter of varying thresholds (*52*). Namely,

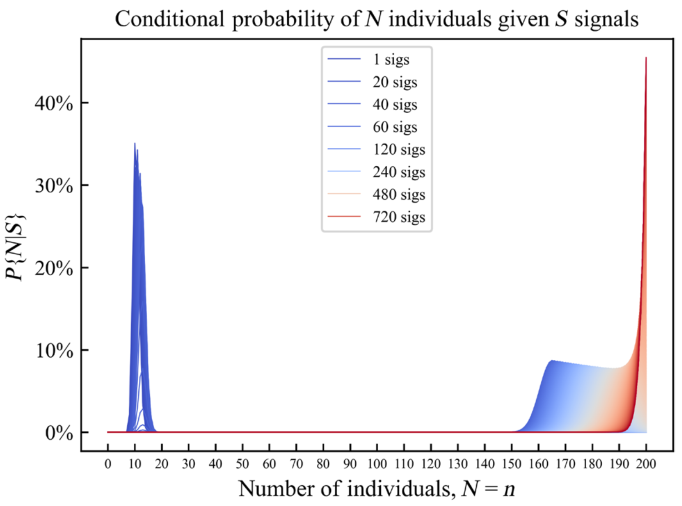

Fig. 3. Semiempirical probability distribution for a number of individuals given a number of GPS signals. In a protected wildlife sanctuary, $\rho_{sanctuary} = 0.523813$.

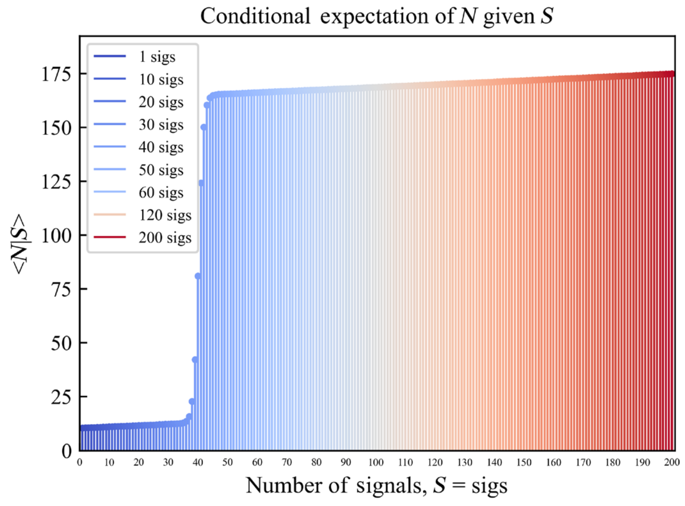

Fig. 4. Conditional expectation of *N* given *S*. In a protected wildlife sanctuary, $\rho_{sanctuary} = 0.523813$, $E_N(s)$.

ERRATUM PUBLISHED 7 FEBRUARY 2025; SEE LAST PAGE

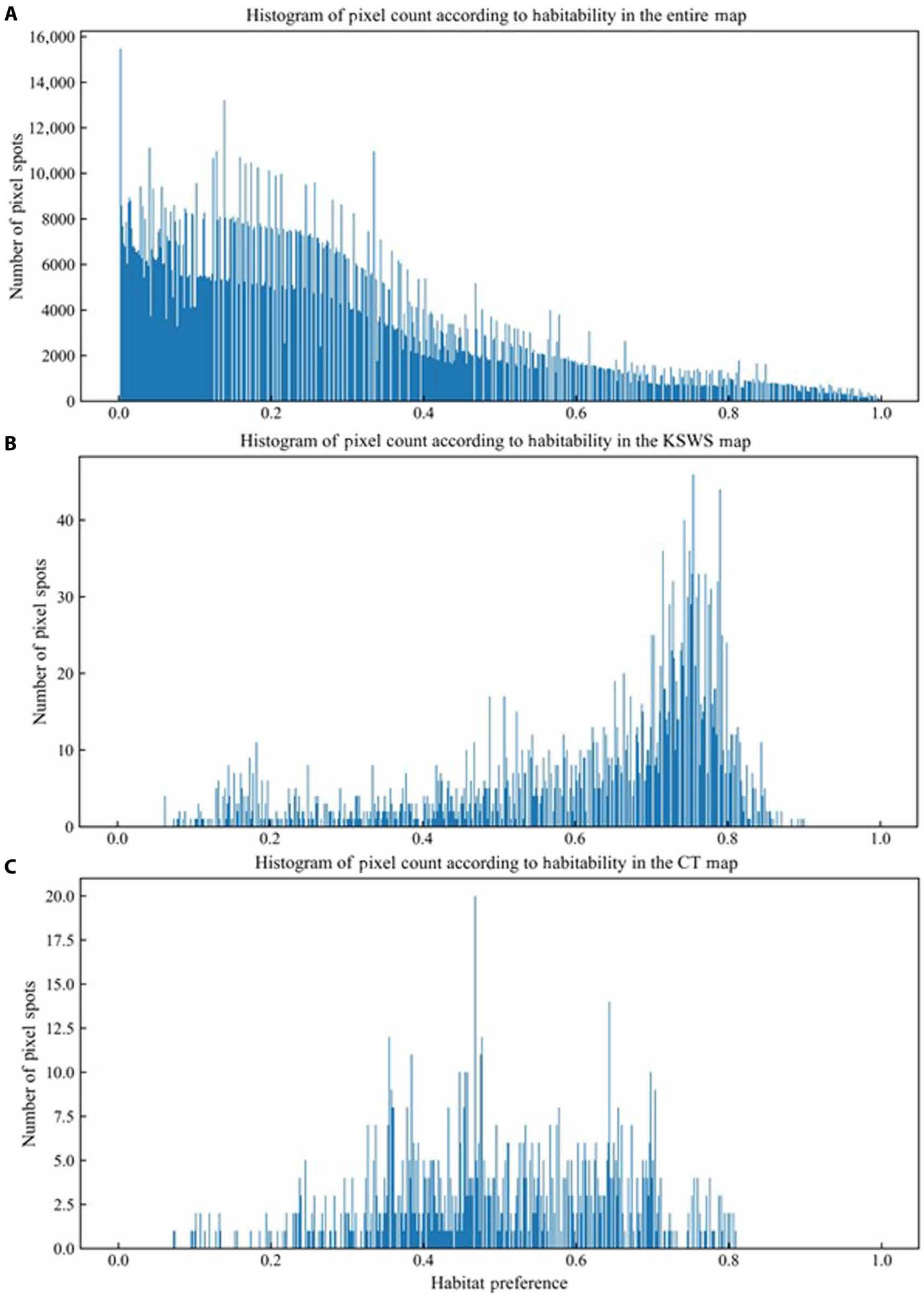

Fig. 5. Histogram of pixel count according to habitat preference. (**A**) Entire habitat preference map. (**B**) KSWS. (**C**) CTNP.

the diffusion values are averaged together with neighboring habitat preference values varying from 0% (no average) to 100% (all values averaged). The possible mean filter thresholds are determined by the resolution of the habitat preference map, spanning 0 to 684 habitat preference values (threshold, $t \in \{0, \ldots, 684\} \in$ Naturals). All possible threshold values were tested and summarized in Fig. 6.

Raw Diffusion of habitat preference h with mean filter threshold t

$$Raw\ Diffusion_h(t) = \frac{\sum_{\frac{-t}{684}}^{\frac{+t}{684}} (Pixel\ Spots_{h+i})}{t} \quad (5)$$

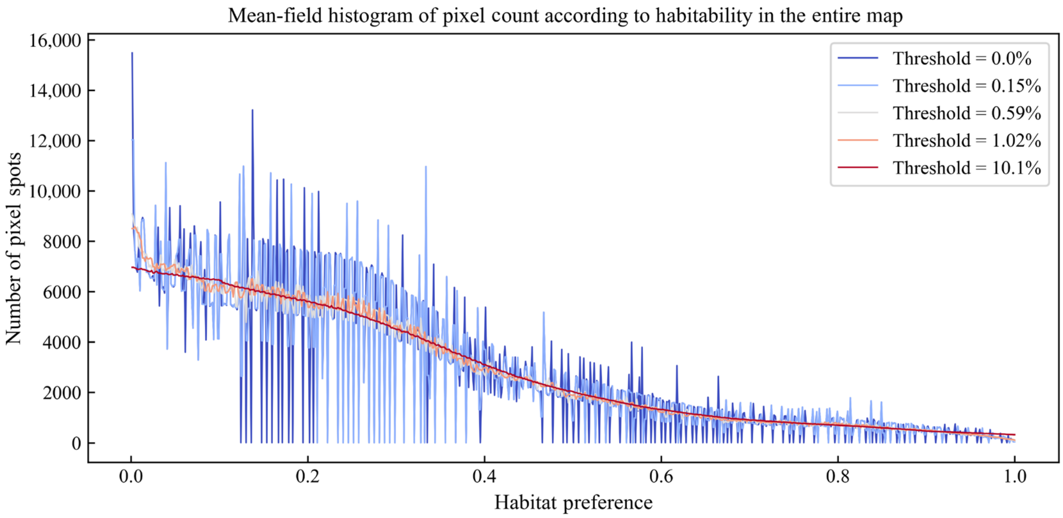

Fig. 6. Selected mean filters for raw diffusion in habitat preference map according to habitat preference thresholds.

where $Pixel\ Spots_h$ = number of pixel spots in the map of habitat preference h.

An empirically corrected (parametrized) diffusion factor is obtained by scaling these raw diffusion factors to reproduce the reported population from Keo Seima, namely

$$Parametric\ Diffusion_h = \frac{(KSWS\ Factor)}{Raw\ Diffusion_h} \tag{6}$$

where $KSWS\ Factor = \frac{(KSWS\ Population)}{\sum_{h \in KSWS}\left(\frac{Individuals_h \cdot Spots_h}{Raw\ Diffusion_h}\right)}$, $KSWS\ population$ = reported population at Keo Seima $\in \{792, 1566, 3097\}$, $\langle Individuals_h \rangle$ = average number of long-tailed macaques at a spot of habitat preference h, $Spots_h$ = number of data spots with habitat preference h, and $Raw\ Diffusion_h = Raw\ Diffusion_h\ (x)$.

This way, the denominator in Eq. 6 represents the abundance of long-tailed macaques in Keo Seima predicted from raw diffusion values alone.

To obtain the final habitat preference–population curves, the expected number of individuals for a pixel spot in the dataset [value obtained from the data signals and the curve for conditional expectation of N, $E_N(s)$, in Fig. 4] is scaled by the parametrized diffusion factor corresponding to its habitat preference; hence

$$Spot\ Population_h = \overline{Individuals_h} \cdot Spots_h \cdot Parametric\ Diffusion_h \tag{7}$$

where $\overline{Individuals_h}$ = Expected number of long-tailed macaque individuals at a pixel spot of habitat preference h.

To ensure continuity in the relationship between habitat preference and population estimation (as well as to incorporate habitat preference values not explicit in the dataset), a mean filter coupled with a univariate spline is applied to the resulting association. All possible mean filter thresholds ($t \in \{0, \ldots, 684\}$) are tested, yielding a four-dimensional tensor (a matrix of matrices, in which two dimensions denote the two mean filter thresholds and two dimensions denote the relation between habitat preference and population). This final tensor is a matrix with 684^2 = 467,856 entries, each entry of which is its own habitat preference–population curve. The mean of the two first dimensions of this tensor is used to obtain a final curve. Given each value of ρ, this process results in three habitat preference–population curves, which can be applied to any region in the habitat preference map.

Supplementary Materials

This PDF file includes:
Supplementary Text
Figs. S1 to S5
Tables S1 and S2
ODMAP Protocol

REFERENCES AND NOTES

1. G. Santini, M. Abolaffio, F. Ossi, B. Franzetti, F. Cagnacci, S. Focardi, Population assessment without individual identification using camera-traps: A comparison of four methods. *Basic Appl. Ecol.* **61**, 68–81 (2022).
2. G. M. Harris, M. J. Butler, D. R. Stewart, E. M. Rominger, C. Q. Ruhl, Accurate population estimation of Caprinae using camera traps and distance sampling. *Sci. Rep.* **10**, 17729 (2020).
3. M. W. Tobler, S. E. Carrillo-Percastegui, R. Leite Pitman, R. Mares, G. Powell, An evaluation of camera traps for inventorying large- and medium-sized terrestrial rainforest mammals. *Anim. Conserv.* **11**, 169–178 (2008).
4. P. Palencia, J. M. Rowcliffe, J. Vicente, P. Acevedo, Assessing the camera trap methodologies used to estimate density of unmarked populations. *J. Appl. Ecol.* **58**, 1583–1592 (2021).
5. D. L. Miller, M. L. Burt, E. A. Rexstad, L. Thomas, Spatial models for distance sampling data: Recent developments and future directions. *Methods Ecol. Evol.* **4**, 1001–1010 (2013).
6. C. C. Sun, J. A. Royle, A. K. Fuller, Incorporating citizen science data in spatially explicit integrated population models. *Ecology* **100**, e02777 (2019).
7. M. T. Farr, D. S. Green, K. E. Holekamp, E. F. Zipkin, Integrating distance sampling and presence-only data to estimate species abundance. *Ecology* **102**, (2021).
8. S. Greenberg, T. Godin, J. Whittington, Design patterns for wildlife-related camera trap image analysis. *Ecol. Evol.* **9**, 13706–13730 (2019).
9. L. Scotson, L. R. Johnston, F. Iannarilli, O. R. Wearn, J. Mohd-Azlan, W. M. Wong, T. N. Gray, Y. Dinata, A. Suzuki, C. E. Willard, Best practices and software for the management and sharing of camera trap data for small and large scales studies. *Remote Sens. Ecol. Conserv.* **3**, 158–172 (2017).
10. Y. Nakashima, K. Fukasawa, H. Samejima, Estimating animal density without individual recognition using information derivable exclusively from camera traps. *J. Appl. Ecol.* **55**, 735–744 (2018).
11. S. T. Buckland, A. J. Plumptre, L. Thomas, E. A. Rexstad, Design and analysis of line transect surveys for primates. *Int. J. Primatol.* **31**, 833–847 (2010).
12. A. J. Plumptre, D. Cox, Counting primates for conservation: Primate surveys in Uganda. *Primates* **47**, 65–73 (2006).

13. M. F. Hansen, V. A. Nawangsari, F. M. van Beest, N. M. Schmidt, A. Fuentes, C. Traeholt, M. Stelvig, T. Dabelsteen, Estimating densities and spatial distribution of a commensal primate species, the long-tailed macaque (*Macaca fascicularis*). *Conserv. Sci. Prac.* **1**, e88 (2019).
14. D. J. I. Seaman, H. Bernard, M. Ancrenaz, D. Coomes, T. Swinfield, D. T. Milodowski, T. Humle, M. J. Struebig, Densities of Bornean orang-utans (*Pongo pygmaeus morio*) in heavily degraded forest and oil palm plantations in Sabah, Borneo. *Am. J. Primatol.* **81**, e23030 (2019).
15. D. E. Bowler, E. B. Nilsen, R. Bischof, R. B. O'Hara, T. T. Yu, T. Oo, M. Aung, J. D. C. Linnell, Integrating data from different survey types for population monitoring of an endangered species: The case of the Eld's deer. *Sci. Rep.* **9**, 7766 (2019).
16. E. J. Talis, C. C. Castaldo, H. J. Lynch, Difficulties in summing log-normal distributions for abundance and potential solutions. *PLOS ONE* **18**, e0280351 (2023).
17. A. A. Eudey, The crab-eating macaque (*Macaca fascicularis*): Widespread and rapidly declining. *Primate Conserv.* **23**, 129–132 (2008).
18. M. F. Hansen, M. Gill, E. F. Briefer, D. R. Nielsen, V. Nijman, Monetary value of live trade in a commonly traded primate, the long-tailed macaque, based on global trade statistics. *Front. Conserv. Sci.* **3**, 16 (2022).
19. L. Santini, S. H. Butchart, C. Rondinini, A. Benítez-López, J. P. Hilbers, A. M. Schipper, M. Cengic, J. A. Tobias, M. A. Huijbregts, Applying habitat and population-density models to land-cover time series to inform IUCN Red List assessments. *Conserv. Biol.* **33**, 1084–1093 (2019).
20. M. N. Nuttall, O. Griffin, R. M. Fewster, P. J. McGowan, K. Abernethy, H. O'Kelly, M. Nut, V. Sot, N. Bunnefeld, Long-term monitoring of wildlife populations for protected area management in Southeast Asia. *Conserv. Sci. Prac.* **4**, e614 (2022).
21. R. Kyes, E. Iskandar, J. Pamungkas, Preliminary survey of the long-tailed macaques (*Macaca fascicularis*) on Java, Indonesia: Distribution and human-primate conflict. *Cambridge Stud. Biol. Evol. Anthropol.* **1**, 65–71 (2011).
22. M. F. Hansen, M. Gill, V. A. Nawangsari, K. L. Sanchez, S. M. Cheyne, V. Nijman, A. Fuentes, Conservation of long-tailed macaques: Implications of the updated IUCN status and the COVID-19 pandemic. *Primate Conserv.* **35**, 1–11 (2021).
23. A. Fuentes, Human culture and monkey behavior: Assessing the contexts of potential pathogen transmission between macaques and humans. *Am. J. Primatol.* **68**, 880–896 (2006).
24. M. D. Gumert, The common monkey of Southeast Asia: Long-tailed macaque populations, ethnophoresy, and their occurrence in human environments, in *Monkeys on the Edge: Ecology and Management of Long-Tailed Macaques and Their Interface with Humans*, M. D. Gumert, L. Jones-Engel A. Fuentes, Eds. (Cambridge, 2011), pp. 3–44.
25. J. Fooden, Systematic review of Southeast Asia longtail macaques, *Macaca fascicularis* (Raffles, 1821). *Fieldiana Zool.* **81**, 1–206 (1995).
26. L. E. Gamalo, K. Ilham, L. J. Engel, M. Gill, R. Sweet, B. Aldrich, P. Phiapalath, T. Van Bang, T. Ahmed, S. Kite, S. Paramasivam, H. Seiha, M. Z. Zainol, D. R. K. Nielsen, N. Ruppert, A. Fuentes, M. F. Hansen, Removal from the wild endangers the once widespread long-tailed macaque. *Am. J. Primatol.* **86**, e23547 (2024).
27. M. D. Gumert, L. Jones-Engel, A. Fuentes, *Monkeys on the Edge: Ecology and Management of Long-Tailed Macaques and Their Interface with Humans* (Cambridge, 2011).
28. NABR, NABR files formal petition challenging the reclassification of long tailed macaques; https://www.nabr.org/about-nabr/news/nabr-files-formal-petition-challenging-reclassification-long-tailed-macaques#:~:text=WASHINGTON%20%E2%80%93%20The%20National%20Association%20for.
29. S. Phillips, A brief tutorial on Maxent. *Lessons Conserv.* **3**, 108–135 (2009).
30. A. Yanuar, The population distribution and abundance of siamangs (*Symphalangus syndactylus*) and agile gibbons (*Hylobates agilis*) in West Central Sumatra, Indonesia, in *The Gibbons: New Perspectives on Small Ape Socioecology and Population Biology*, D. Whittaker, S. Lappan, Eds. (Springer, 2009), pp. 453–465.
31. M. Bessone, H. S. Kühl, G. Hohmann, I. Herbinger, K. P. N'Goran, P. Asanzi, P. B. Da Costa, V. Dérozier, D. B. E. Fotsing, B. B. Ikembelo, D. M. Iyomi, B. I. Iyatshi, P. Kafando, A. M. Kambere, B. D. Moundzoho, L. K. Musubaho, B. Fruth, Assessing the effects of survey-inherent disturbance on primate detectability: Recommendations for line transect distance sampling. *Primates* **64**, 107–121 (2023).
32. J. Fooden, Comparative review of fascicularis-group species of macaques (Primates: Macaca). *Fieldiana Zool.* **107**, 1–43 (2006).
33. K. MacKinnon, K. MacKinnon, Conservation status of primates in Malaisia, with special reference to Indonesia. *Primate Conserv.* **8**, 175–183 (1987).
34. J. H. Moore, L. Gibson, Z. Amir, W. Chanthorn, A. H. Ahmad, P. A. Jansen, C. P. Mendes, M. Onuma, C. A. Peres, M. S. Luskin, The rise of hyperabundant native generalists threatens both humans and nature. *Biol. Rev.* **98**, 1829–1844 (2023).
35. M. F. Hansen, A. Ang, T. Trinh, E. Sy, S. Paramasiwam, T. Ahmed, J. Dimalibot, L. Jones-Engel, N. Ruppert, C. Griffioen, N. Lwin, P. Phiapalath, R. Gray, S. Kite, N. Doak, V. Nijman, A. Fuentes, M. D. Gumert, *Macaca fascicularis* (amended version of 2022 assessment). The IUCN Red List of Threatened Species 2022: e.T12551A221666136 (2023).
36. CITES, Convention on International Trade in Endangered Species of Wild Fauna and Flora; https://cites.org/sites/default/files/eng/com/sc/66/E-SC66-31-01.pdf.
37. K. Karuppannan, S. Saaban, A. Mustapa, F. Zainal Abidin, N. Azimat, C. Keliang, Population status of long-tailed macaque (*Macaca fascicularis*) in Peninsular Malaysia. *J. Primatol.* **3**, 34–37 (2014).
38. PERHILITAN, Laporon Ketahunan (Annual Report) 2018. Department of Wildlife and National Parks Peninsular Malaysia (2018).
39. D. Zurell, J. Franklin, C. König, P. J. Bouchet, C. F. Dormann, J. Elith, G. Fandos, X. Feng, G. Guillera-Arroita, A. Guisan, J. J. Lahoz-Monfort, P. J. Leitão, D. S. Park, A. T. Peterson, G. Rapacciuolo, D. R. Schmatz, B. Schröder, J. M. Serra-Diaz, W. Thuiller, K. L. Yates, A standard protocol for reporting species distribution models. *Ecography* **43**, 1261–1277 (2020).
40. T. L. Graham, H. D. Matthews, S. E. Turner, A global-scale evaluation of primate exposure and vulnerability to climate change. *Int. J. Primatol.* **37**, 158–174 (2016).
41. S. E. Fick, R. J. Hijmans, WorldClim 2: New 1-km spatial resolution climate surfaces for global land areas. *Int. J. Climatol.* **37**, 4302–4315 (2017).
42. J. A. Grytnes, Species-richness patterns of vascular plants along seven altitudinal transects in Norway. *Ecography* **26**, 291–300 (2003).
43. The Ministry of Economy, Trade, and Industry (METI) of Japan and the United States National Aeronautics and Space Administration (NASA), ASTER Global Digital Elevation Map; https://asterweb.jpl.nasa.gov/gdem.asp.
44. N. Rowe, *The Pictorial Guide to the Living Primates* (Pogonias, 1996).
45. S. J. Phillips, R. P. Anderson, R. E. Schapire, Maximum entropy modeling of species geographic distributions. *Ecol. Model.* **190**, 231–259 (2006).
46. S. J. Phillips, M. Dudík, R. E. Schapire, A maximum entropy approach to species distribution modeling, in *ICML '04: Proceedings of the Twenty-First International Conference on Machine Learning* (Association for Computing Machinery, 2004).
47. J. Swets, Measuring the accuracy of diagnostic systems. *Science* **240**, 1285–1293 (1988).
48. M. F. Hansen, S. Ellegaard, M. M. Moeller, F. M. Van Beest, A. Fuentes, V. A. Nawangsari, C. Groendahl, M. L. Frederiksen, M. Stelvig, N. M. Schmidt, Comparative home range size and habitat selection in provisioned and non-provisioned long-tailed macaques (*Macaca fascicularis*) in Baluran National Park, East Java, Indonesia. *Contrib. Zool.* **89**, 393–411 (2020).
49. J. C. M. Sha, M. D. Gumert, B. P. Y. H. Lee, A. Fuentes, S. Rajathurai, S. Chan, L. J. Engel, Status of the long-tailed macaque *Macaca fascicularis* in Singapore and implications for management. *Biodivers. Conserv.* **18**, 2909–2926 (2009).
50. J. Wäldchen, P. Mäder, Machine learning for image based species identification. *Methods Ecol. Evol.* **9**, 2216–2225 (2018).
51. O. H. Diserud, S. Engen, A general and dynamic species abundance model, embracing the lognormal and the gamma models. *Am. Nat.* **155**, 497–511 (2000).
52. F. Kienberger, V. Pastushenko, G. Kada, T. Puntheeranurak, L. Chtcheglova, C. Riethmueller, C. Rankl, A. Ebner, P. Hinterdorfer, Improving the contrast of topographical AFM images by a simple averaging filter. *Ultramicroscopy* **106**, 822–828 (2006).

Acknowledgments: We are grateful to the immense magnitude of individuals, organizations, and institutions that supported this research, especially all the local communities involved in fieldwork. We thank the Long-Tailed Macaque Project, in particular A. Mckay, J. Movva, M. S. Larsen, and N. R. Krog, whose volunteer collection and analysis of pictures of long-tailed macaques in social media prompted invaluable discussions of the reported sighting data. We are also grateful to the Department of Anthropology, Princeton University, USA for support. The research herein uses data from the Wildlife Conservation Society (WCS). The views expressed here are those of the author(s) and do not necessarily represent the views of WCS or other data contributors. Any errors are attributable to the author(s). Survey in Preah Vihear Protected Area jointly conducted by AS and WCS Cambodia was financially supported by the Point Defiance Zoo & Aquarium's Conservation Fund, Sasakawa Scientific Research Grant from the Japan Science Society, the JASSO Short Stay Program, Kyoto University Inter-Graduate School Program for Sustainable Development and Survivable Societies, and a Grant-in-Aid for JSPS Research Fellow (grant number 16J08359). We are grateful to the Forestry Administration of Cambodia. We credit WWF-Cambodia, the Mondulkiri Provincial Department of Environment (PDoE), and the Ministry of Environment (MoE) and the Forestry Administration (FA) of the Royal Government of Cambodia (RGC) as well as co-authors affiliated therewith for the contributions to this project. Camera trap data collected by the Wildlife Conservation Research Unit (WildCRU) and/or Panthera Corporation, a registered nonprofit organization, in SWS and/or PPWS during 2013, 2014, 2016, 2018, 2019, and 2021 comprise datasets co-owned by WildCRU and/or Panthera, which contributed funding to surveys and collection of the data used herein. Wildlife Conservation Research Unit was provided funding by the Robertson Foundation and the Panthera Leopard Program. We are grateful to all the field personnel who helped conduct the camera trap survey. University of Liège, LTMP Funding was provided by the Belgian National Fund for Scientific Research (FNRS). Research was conducted in full compliance with Vietnamese regulations and was carried out under research permission granted by the Cát Tiên National Park and the Dong Nai Province (work permit no. 075851). Fishing Cat Ecological Enterprise (FCEE) funding was provided by Mohamed bin Zayed Species Conservation Fund, Panthera Small Cats Program, and Fishing Cat Conservation Alliance. We are grateful to the Ministry of Environment, Koh Kong Provincial Department of Environment

and local rangers and community members for their ongoing collaboration. Bangladesh Forest Department provided research permission to conduct the field survey on the southeastern coast of Bangladesh, while Nature Conservation Management (NACOM), Isabela Foundation, and Bangladesh Border Guard (BGB) supported the fieldwork in many ways. This article uses camera trap data collected under the US Agency for International Development (USAID) Greening Prey Lang (GPL). USAID GPL was led by Tetra Tech with support from two major partners: WCS and Conservation International. Camera trap surveys were financed by USAID/Cambodia under the USAID Greening Prey Lang (GPL) Activity in four protected areas: Chhaeb Wildlife Sanctuary, Preah Roka Wildlife Sanctuary, Phnom Tbaeng Natural Heritage Park, and Prey Lang Wildlife Sanctuary. The author's views expressed in this publication do not necessarily reflect the views of the USAID or the US government. The Long-Tailed Macaque Project is grateful to all their funders, especially Animal Protection Denmark. We are grateful to People for the Ethical Treatment of Animals for supporting the off-site data analysis that supported the model. M.F.H. is beyond grateful to Re:wild and the Carlsberg Foundation Internationalization Fellowship grant (CF21-0473). **Funding:** This work was supported by the WildCRU, Panthera, Robertson Foundation, Panthera Leopard Program, Belgian National Fund for Scientific Research (FNRS), Mohamed bin Zayed Species Conservation Fund, Panthera Small Cats Program, Fishing Cat Conservation Alliance, Sasakawa Scientific Research Grant from the Japan Science Society, USAID, Point Defiance Zoo & Aquarium's Conservation Fund, the JASSO Short Stay Program, Kyoto University Inter-Graduate School Program for Sustainable Development and Survivable Societies, Grant-in-Aid for JSPS Research Fellow, Animal Protection Denmark, Re:wild, Carlsberg Foundation (grant CF21-0473), People for the Ethical Treatment of Animals, and Department of Anthropology, Princeton University. **Author contributions:** Conceptualization: M.F.H., A.L.K.L., A.F., and X.Z. Model methodology: M.F.H., A.L.K.L., A.F., X.Z., and D.R.K.N. Field methodology: C.A., T.V.B., S.A., E.A., E.G., J.L.F., J.F.K., K.I., Tanvir Ahmed, M.G., N.R., N.H., N.C., N. Lw., N. Le., P.S., P.P., S.H., S.B., S.B.-E., S.N., S.L., Toufique Ahmed, V.A., V.H.M., and Z.Z. Model investigation: M.F.H., A.L.K.L., and X.Z. Field investigation: A.A., T.V.B., C.A., S.A., E.A., E.G., J.L.F., J.F.K., K.I., Tanvir Ahmed, M.G., N.R., N.H., N.C., N. Lw., N. Le., P.S., P.P., S.H., S.B., S.B.-E., S.N., S.L., Toufique Ahmed, V.A., V.H.M., and Z.Z. Visualization: A.L.K.L. and X.Z. Supervision: M.F.H., A.F., and E.F.B. Writing—original draft: M.F.H., A.L.K.L., and X.Z. Writing—review and editing: All authors. **Competing interests:** The authors declare that they have no competing interests. **Data and materials availability:** All data needed to evaluate the conclusions in the paper are present in the paper and/or the Supplementary Materials. Sightings' data, python scripts, and plots are available on the repository Zenodo (https://doi.org/10.5281/zenodo.10903272).

Submitted 15 December 2023
Accepted 22 April 2024
Published 24 May 2024
10.1126/sciadv.adn5390

Erratum

Post date 7 February 2025

Erratum for the Research Article: "A model for the noninvasive, habitat-inclusive estimation of upper limit abundance for synanthropes, exemplified by *M. fascicularis*" by A. L. Koch Liston *et al.*

The original version of the Research Article "A model for the noninvasive, habitat-inclusive estimation of upper limit abundance for synanthropes, exemplified by *M. fascicularis*" by A. L. Koch Liston *et al*. has been updated to reflect a revised author list.

The following corrections have been implemented:

- Seiha Hun, Neth Vibol, and Sophatt Reaksmey have been removed from the author list.

The author list, affiliations, and author contributions sections of the Acknowledgements have been corrected to reflect these changes in the updated PDF and HTML versions of the article.

10.1126/sciadv.adw3899
