# Supplementary material for: A model for the noninvasive, habitat-inclusive estimation of upper limit abundance for synanthropes, exemplified by M. fascicularis
Source: Sci Adv. 2024 May 24;10(21):eadn5390. doi: 10.1126/sciadv.adn5390 (PMC11122667; doi:10.1126/sciadv.adn5390)
Supplement: Supplementary file 3 — Supplementary Text Figs. S1 to S5 Tables S1 and S2 ODMAP Protocol [file sciadv.adn5390_sm.pdf]

Science Advances
AAAS

# Supplementary Materials for

## A model for the noninvasive, habitat-inclusive estimation of upper limit abundance for synanthropes, exemplified by *M. fascicularis*

André L. Koch Liston *et al.*

Corresponding author: Malene F. Hansen, malenefriishansen@gmail.com

*Sci. Adv.* **10**, eadn5390 (2024)
DOI: 10.1126/sciadv.adn5390

**This PDF file includes:**

Supplementary Text
Figs. S1 to S5
Tables S1 and S2
ODMAP Protocol

**Supplementary Text**

**Explore Data, Scripts, and Plots on GitHub**

All sightings' data, python scripts and plots are available here: TheLTMProject. (2024). TheLTMProject/Upper-Limit-Estimation-for-Wildlife-Abundance: Zenodo Integration (Main). Zenodo. https://doi.org/10.5281/zenodo.10903272

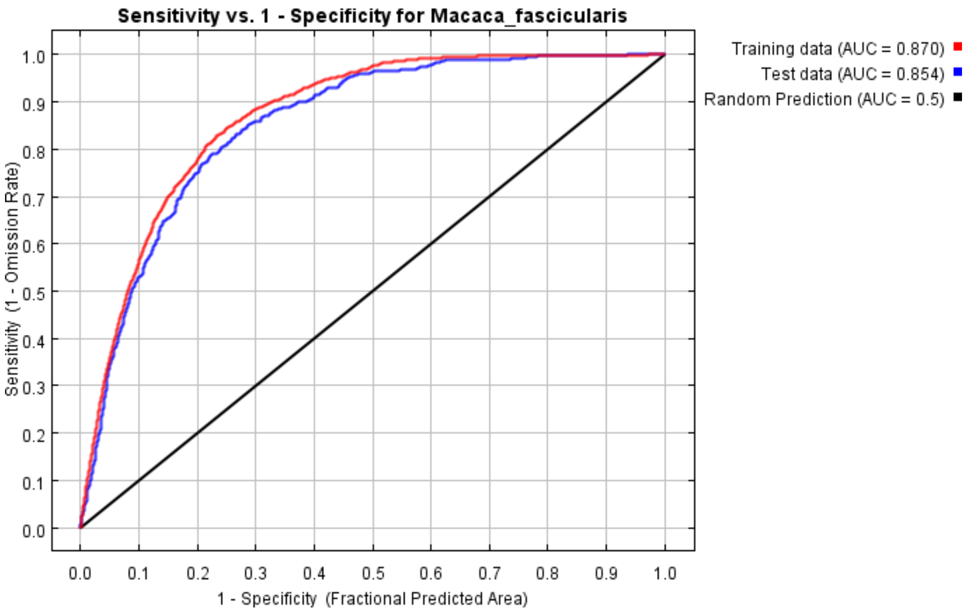


**Fig. S1. ROC curve of the predicted result of the MaxEnt model**. The red (training) line shows the "fit" of the model to the training data, while the blue (testing) line indicates the fit of the model to the testing data.

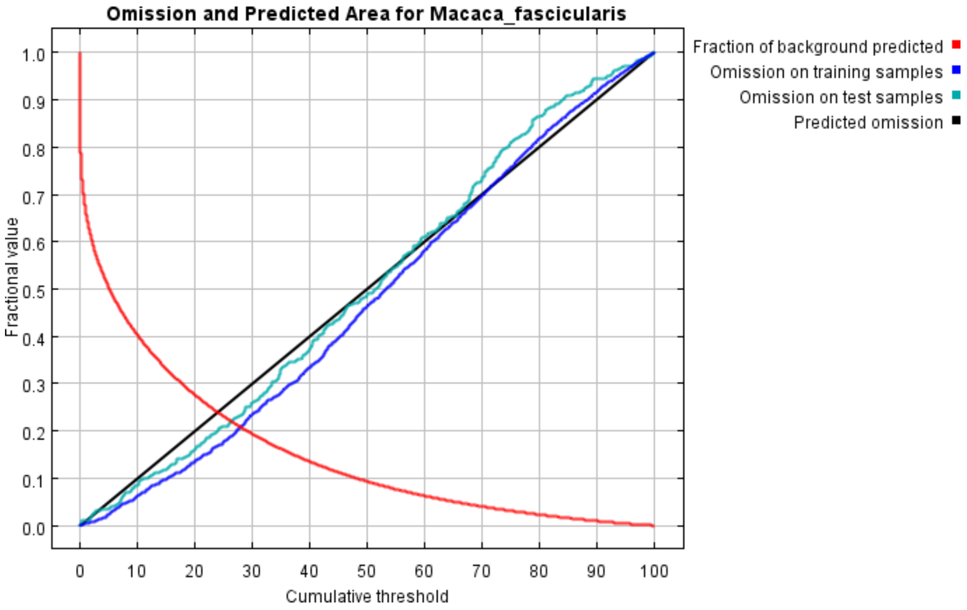


**Fig. S2. Omission rates for the output of the MaxEnt model**. The figure shows how testing and training omission and predicted area vary with the choice of cumulative threshold.

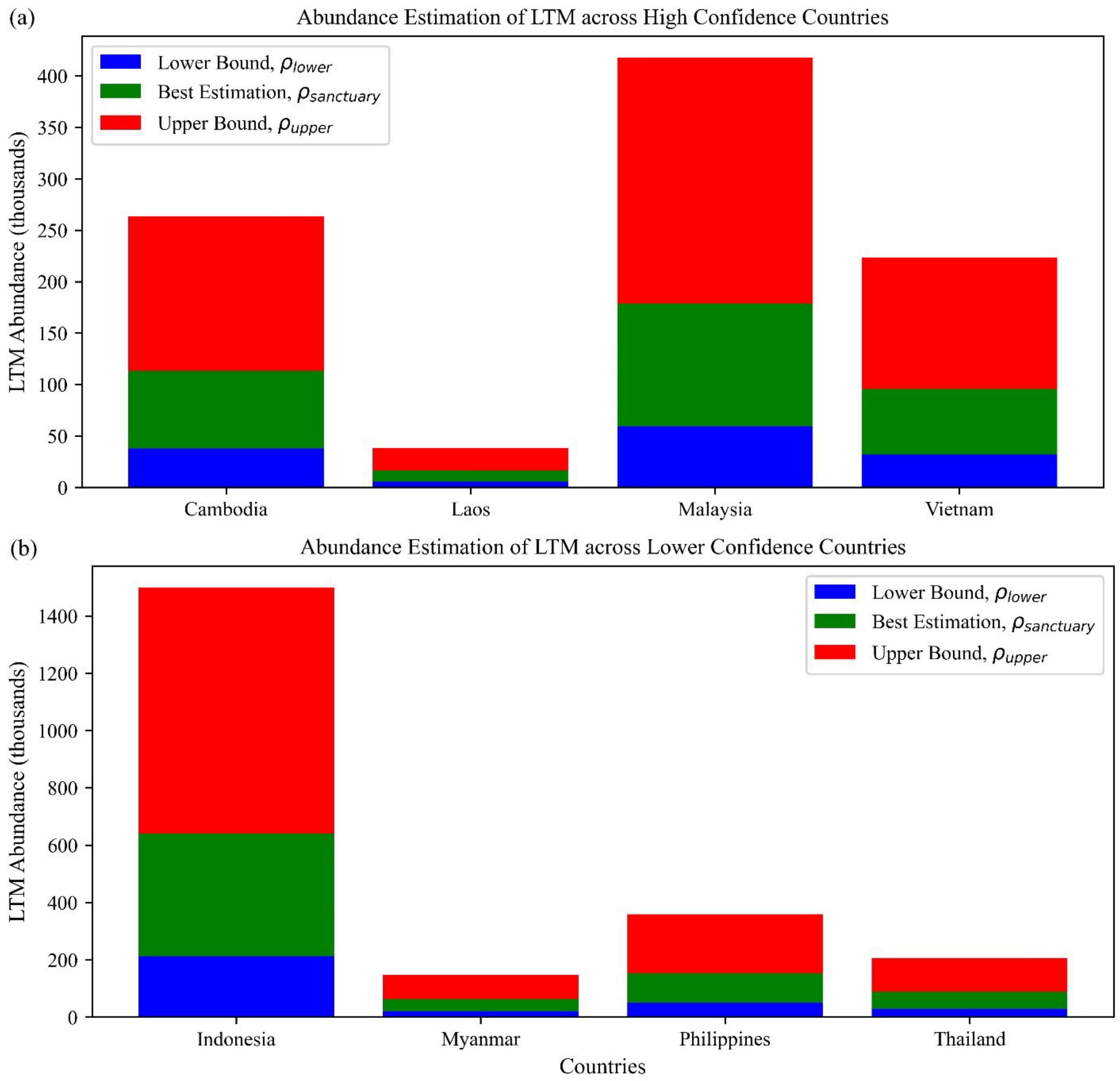


**Fig. S3. Model Estimation for the Upper Limit long-tailed macaque Abundance**. (A) High Confidence Countries (Cambodia, Laos, Malaysia, and Vietnam), and (B) Lower Confidence

Countries (Indonesia, Myanmar, Philippines, and Thailand) according to parametrization from a protected Wildlife Sanctuary, $\rho_{sanctuary}$.

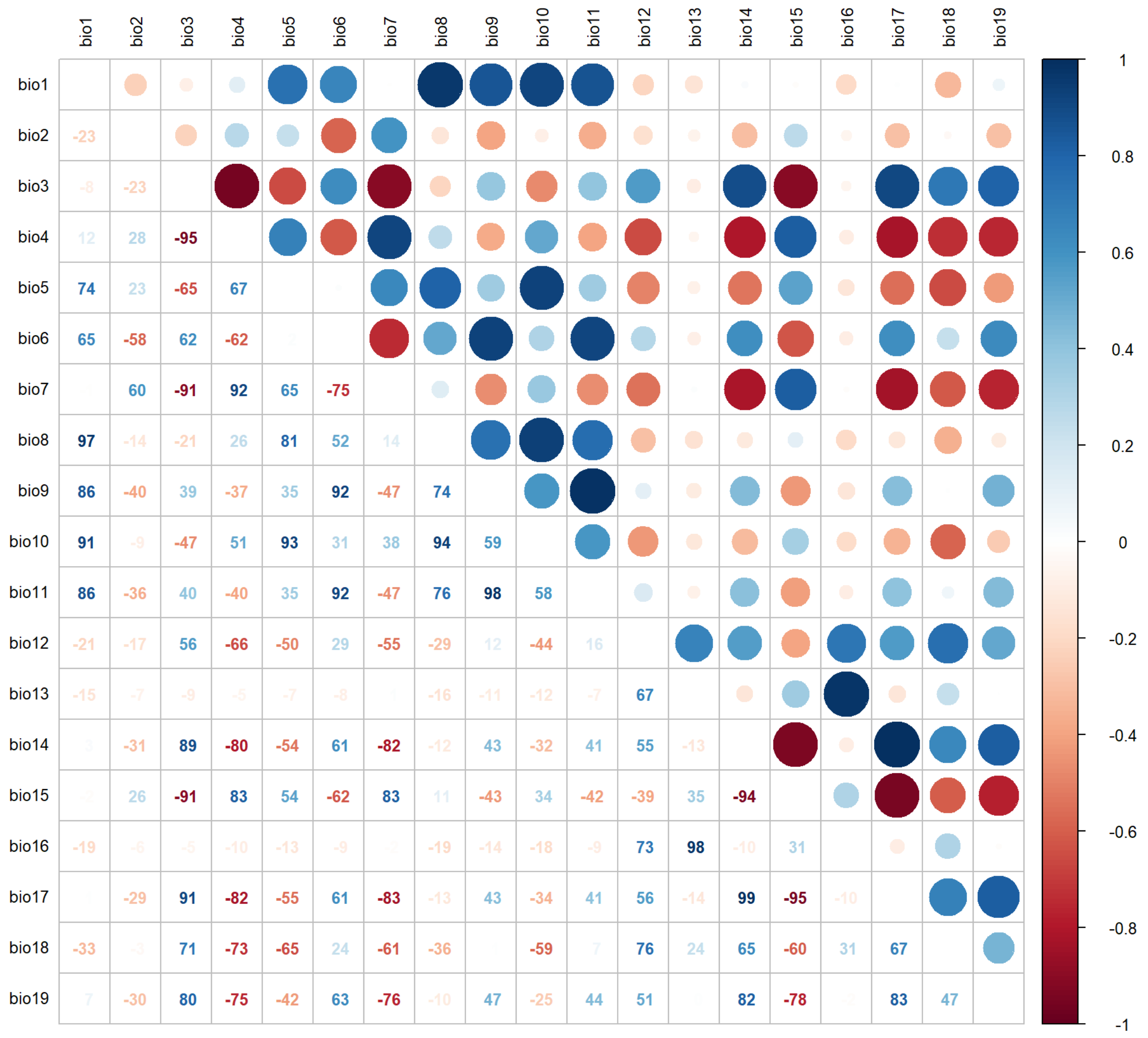


**Fig. S4. Correlation plot of 19 bioclimatic factors under the data of long-tailed macaque distribution**. Correlations are displayed using color, circle diameter, and numerical values. Larger absolute values and diameters, as well as darker colors, indicate higher correlations, while smaller absolute values and diameters, as well as lighter colors, represent weaker correlations.

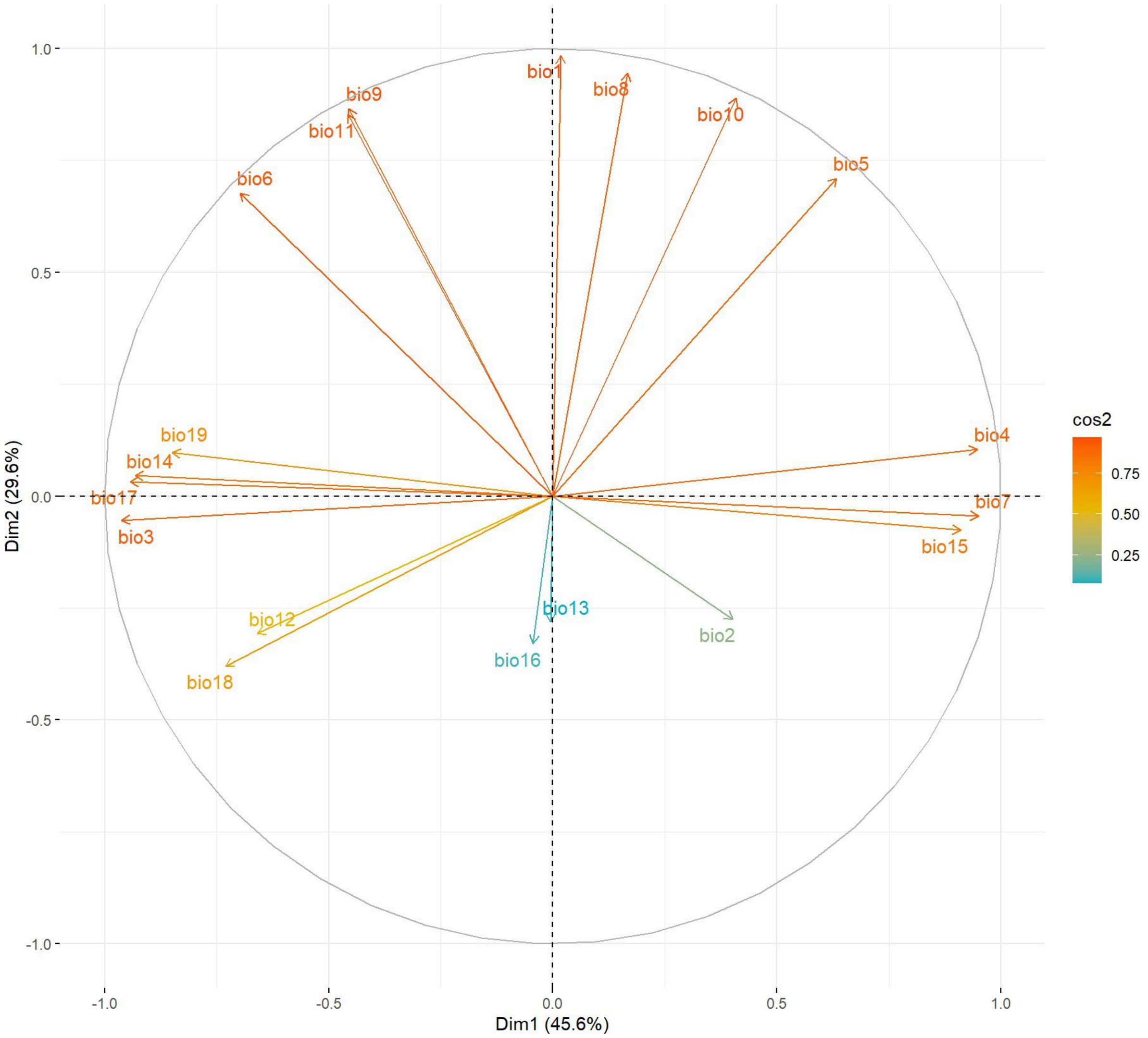


**Fig. S5. Pie chart of PCA of 19 bioclimatic factors illustrating their impact on the distribution of long-tailed macaques**. The cos2 values represent the contribution of variables to the principal components. A larger cos2 value corresponds to a more significant contribution, indicated by a red color; conversely, a smaller cos2 value, indicated by a blue color, corresponds a lower contribution. The horizontal and vertical axes depict the two dimensions of variable distribution in the principal component space, while the arrow angles illustrate the contribution of variables along these two dimensions.

**Table S1.** Signal Sources sorted by Country. Data signals denote the total number of long-tailed macaque individuals reported across all reported sightings in the source.

| **Country**<br>- Source | **Sightings** | **Data Signals** |
|---|---|---|
| **Cambodia** | **2,229** | **5,473** |
| - CI | 22 | 22 |
| - FII | 6 | 6 |
| - FCEE | 86 | 331 |
| - iNaturalist | 124 | 124 |
| - USAID | 1167 | 1,587 |
| - WCS-GBIF 'Long-term monitoring of primate, bird, and ungulate populations 2010-2022 for protected area management, Keo Seima Wildlife Sanctuary, Cambodia' | 67 | 249 |
| - WCS-SMART | 551 | 2,780 |
| - Wildlife Alliance | 34 | 202 |
| - WWF | 172 | 172 |
| **Indonesia** | **124** | **4,910** |
| - iNaturalist | 730 | 730 |
| - Direct Observation by Kurnia (the Long-Tailed Macaque Project, Andalas University) | 49 | 2,195 |
| **Laos** | **341** | **837** |
| - Direct Observation by Phiapalath (the Long-tailed Macaque project) | 340 | 836 |
| **Malaysia** | **7,873** | **7,873** |
| - iNaturalist | 670 | 670 |

| | | |
|---|---|---|
| - **[Not Used in Habitat-Preference Map]** Malaysia Wildlife Department (Zainol, Universiti Sains Malaysia) | 7,203 | 7,203 |
| **Myanmar** | **13** | **1,228** |
| - Direct Observation by Lwin (Flora and Fauna International Myanmar) | 13 | 1,228 |
| **Philippines** | **133** | **133** |
| - iNaturalist | 133 | 133 |
| **Singapore** | **988** | **988** |
| - iNaturalist | 988 | 988 |
| **Thailand** | **386** | **691** |
| - Direct Observation by Challis (Neil Challis Photography) | 10 | 315 |
| - iNaturalist | 376 | 376 |
| **Vietnam** | **124** | **763** |
| - Direct Observation by Gazagne & Alexiadou (University of Liège, University of Copenhagen and the Long-Tailed Macaque Project) | 16 | 16 |
| - iNaturalist | 69 | 69 |
| - Direct Observation by Bang (Southern Institute of Ecology) | 26 | 564 |
| - Direct Observation by Gazagne | 13 | 114 |
| **Total** | **12,867** | **20,912** |

**Table S2.** Effect of Inquisitiveness ρ on Population Estimate from Semi-empirical Probability Model in Keo Seima Wildlife Sanctuary and Cát Tiên National Park.

| **Inquisitiveness Factor ρ** | 0 | 0.523813 | 1 |
|---|---|---|---|
| **Reported Population (10)** | 792 | 1566 | 3097 |
| **Model Population for KSWS** | 854 | 1566 | 3097 |
| **Signals Representing Non-repeated Individuals** | 9.6% | 29.33% | 100% |
| **Model Population for CTNP** | 177 | 806 | 830 |
| **Signals Representing Non-repeated Individuals** | 9.60% | 29.27% | 100% |

**ODMAP**

# A New Model for Non-invasive, Habitat-Inclusive Estimation of Upper Limit Abundance for Synanthropes, Exemplified by *M. fascicularis*

## – ODMAP Protocol –

André L. Koch Liston, Xueying Zhu, Bang V. Tran, Phaivanh Phiapalath, Seiha Hun, Tanvir Ahmed, Sabit Hasan, Sajib Biswas, Shimul Nath, Toufique Ahmed, Kurnia Ilham, Ngwe Lwin, Jackson L. Frechette, Naven Hon, Cain Agger, Suzuki Ai, Emeline Auda, Eva Gazagne, Jan F. Kamler, Milou Groenenberg, Sarah Banet-Eugene, Neil Challis, Neth Vibol, Nicole Leroux, Pablo Sinovas, Sophatt Reaksmey, Vanessa H. Muñoz, Susan Lappan, Zaki Zainol, Valeria Albanese, Athanasia Alexiadou, Daniel RK Nielsen, Anna Holzner, Nadine Ruppert, Elodie Briefer, Agustin Fuentes, Malene F. Hansen

2024-03-13

---

## Overview

*Authorship*

Contact : malenefriishansen@gmail.com, koch.liston@gmail.com, xueying.zhu@research.uwa.edu.au

Study link: Under Review

*Model objective*

Model objective: Mapping and interpolation.

Target output: Habitat-preference map and abundance population.

*Focal Taxon*

Focal Taxon: Synanthrope.

*Location*

Location: Southeast Asia.

*Scale of Analysis*

Spatial extent: 88.080826980, 129.881559354, 25.157063354, -14.288698182 (xmin, xmax, ymin, ymax)

Spatial resolution: 1.143 $km^2$

Temporal extent: January 2020 through December 2022.

Temporal resolution: Day.

Boundary: Political.

*Biodiversity data*

Observation type: GPS tracking, field survey, citizen science, range map.

Response data type: Counts, Point occurrence.

*Predictors*

Predictor types: Habitat.

*Hypotheses*

Hypotheses: Average group size and density of species are hypothesized to be strictly increasing with habitat-preference.

*Assumptions*

Model assumptions: Assumes the region of interest to obey a dispersion between habitat-preference and average group size similar to the region of calibration – in this case, Keo Seima Wildlife Sanctuary.

*Algorithms*

Modelling techniques: Maxent and occupancy.

Model complexity: MaxEnt is used to derive habitat-preference maps, which are then combined with occupancy data to estimate the relation between habitat-preference and group-size and abundance.

Model averaging: A mean-value filter combines into a final average of all possible cutoff thresholds for the distributions relating habitat-preference to average group-size; this allows the

extrapolation of expected group-sizes for habitat-preference values without direct, empirical data.

*Workflow*

Model workflow: Geographical location of species occurrence is used in MaxEnt to obtain a habitat-preference map for the region of interest. Given a calibration site, for which both habitat-preference values and species occurrence is known, the model generates a fit between habitat-preference and GPS signals. This results in a habitat-sensitive prediction of population abundance to the region of interest with minimal starting data.

*Software*

Software: MaxEnt, QGIS, ArcGIS, R, Python.

Code availability: Example usage is available at https://github.com/TheLTMProject/Upper-Limit-Estimation-for-Wildlife-Abundance/tree/main

Data availability: Example data sets for *M. fascicularis* are available at https://drive.google.com/drive/folders/16MRhrhz6oxsYEaj-d8UkgaXIZA2d9nuT?usp=sharing

## Data

*Biodiversity data*

Taxon names: *Macaca fascicularis* (long-tailed macaque)

Taxonomic reference system: GBIF Backbone Taxonomy.

Ecological level: Populations.

Data sources: iNaturalist public database, camera traps, transects, and direct observations from co-authors.

Sampling design: Countries of interest (Cambodia, Indonesia, Laos, Malaysia, Myanmar, Philippines, Singapore, Thailand, Vietnam) across temporal extent.

Sample size: 20,912 total individuals reported from 12,867 GPS signals.

Clipping: Due to extremely limited data sets, no masks were applied.

Scaling: N/A. GPS signals offer accuracy way below resolution threshold of mapped area (60 m << 1 km).

Cleaning: Co-author collaborators validated species identification. Signals closer than 800 m (home-range of species) were grouped as one single GPS source.

Absence data: Absence data not available for species.

Background data: Minimum and maximum limits for group size must be specified. If species shows more than one sort of group (e.g. provisioned vs non-provisioned), the expected ratios of groups must be specified. Mean and standard deviation of number of individuals in groups must be specified. For this study, this information was acquired through local specialists, many of them co-authors, as well as literary research.

Errors and biases: Abundance estimation relies strongly on calibration using a mapped region. In this example, Keo Seima Wildlife Sanctuary, a protected region was used, which contributes to an overestimation of the final population abundance.

*Data partitioning*

Training data: Twenty percent of data used as training data in MaxEnt.

Validation data: The MaxEntmodel in this study was built without the use of validation data. Due to certain limitations, we were unable to assess the model's generalization performance. A surface evaluation of the model's validity was performed using data from Cát Tiên National Park, but – because this region lacks a proper abundance estimation – this test serves only as sanity check for the model outside calibration site.

Test data: Eighty percent of data used as testing data in MaxEnt.

*Predictor variables*

Predictor variables: Annual mean temperature, isothermality, temperature seasonality, mean temperature of wettest Quarter, precipitation of wettest month, precipitation seasonality, elevation, aspect, slope, built areas, crop lands, grasslands, shrublands, forests, distance to the coastline, and distance to roads.

Data sources: WorldClim, EarthEnv, Topography Advanced Spaceborne Thermal Emission and Reflection Radiometer (ASTER), Copernicus Global Land Service, Natural Earth, and Global Roads.

Spatial extent: 92.208333333, 21.174999999, 127.299999999, -10.358333333 (xmin, xmax, ymin, ymax)

Spatial resolution: 1 $km^2$

Coordinate reference system: EPSG:4326

Temporal extent: 1980 to 2020.

Temporal resolution: 30 s.

Data processing: Uniformed layer boundaries, coordinates, and grid sizes.

Dimension reduction: N/A.

*Transfer data*

Data sources:

Spatial extent: N/A.

Spatial resolution: N/A.

Temporal extent: N/A.

Models and scenarios: N/A.

Quantification of Novelty: N/A.

## Model

*Variable pre-selection*

Variable pre-selection: Environmental variables used for MaxEnt were selected based on the experiential criteria and behavior of the long-tailed macaque.

*Multicollinearity*

Multicollinearity: Bioclimatic variables underwent PCA screening to avoid the repetition of variables influencing habitat preferences within the same dimension in the Maxent model.

*Model settings*

<maxent>: The 'Apply threshold rule' was set to '10 percentile training presence' and retained default values for other parameters. The output format specified as cloglog.

Model settings (extrapolation):  N/A.

*Model estimates*
Coefficients: ρ (inquisitiveness) = 0.523813

Parameter uncertainty: If multiple calibration locations are available, a Bayesian correction may be applied to average out inquisitiveness values and obtain an associated uncertainty.

Variable importance: Bioclimatic variables were subjected to PCA screening to identify the most influential variables affecting habitat preferences.

*Model selection - model averaging - ensembles*

Model selection: Information-theoretic approach.

Model averaging: Mean-field average assigns no particular weight to any single threshold; it is expected that weighing becomes necessary in case a strong discrepancy emergers between the average result curve and any one particular curve – this can be easily computed by a normalized distance of squares.

Model ensembles: We explored various combinations of environmental variables, model boundaries, and model parameter settings to identify the optimal model. At the end, only the reported combination was used.

*Analysis and Correction of non-independence*

Spatial autocorrelation: Given the extensive study area, limited data quantity, and diverse data gathering methods, spatial autocorrelation has not been taken into consideration in this model.

Temporal autocorrelation: N/A.

Nested data: N/A.

*Threshold selection*

Threshold selection: 10% training presence.

## Assessment

*Performance statistics*

Performance on training data: AUC (0.870)

Performance on validation data: N/A.

Performance on test data: AUC (0.854)

*Plausibility check*

Response shapes: Consistent with expectations.

Expert judgement: N/A.

## Prediction

*Prediction output*

Prediction unit: Estimated number of individuals in the region of interest.

Post-processing: Already contained in algorithm – Mean-field average of expected number of individuals according to habitat-preference for all cutoff thresholds.

*Uncertainty quantification*

Algorithmic uncertainty: The model carries no stochastic behavior and has a bijective mapping to outputs.

Input data uncertainty: Due to the predictive nature of the model, any biases in the input data will be magnified in extrapolations to larger regions.

Parameter uncertainty: Due to the predictive nature of the model, any biases in the input parameters will be magnified in extrapolations to larger regions.

Scenario uncertainty: Final population uncertainty emerges directly from calibration region, given as the root of individual counts for the given area under investigation.

Novel environments: Accuracy is proportional to the total area and diversity of calibration regions. For small data sets in which this is not available, the limiting cases of $\rho = 0$ and $\rho = 1$ serve as obtain limits for the behavior of the final population estimate.
